# Atrial Fibrillation Ablation: Impact on Burden and Cardiovascular Outcomes

**DOI:** 10.3390/jcm14082648

**Published:** 2025-04-12

**Authors:** Ahmed AlTurki, Vidal Essebag

**Affiliations:** 1 Division of Cardiology, McGill University Health Center, Montreal, QC H3G 1A4, Canada; 2 Department of Medicine, Faculty of Medicine, Kuwait University, Jabriya 13009, Kuwait; 3 Hôpital Sacré-Coeur de Montréal, Montreal, QC H4J 1C5, Canada

**Keywords:** atrial fibrillation, catheter ablation, burden

## Abstract

Atrial fibrillation [AF] is the most common sustained arrhythmia observed in clinical practice with considerable cardiovascular morbidity and mortality. AF burden provides a quantitative measurement of AF and is now more readily achievable utilizing wearable and implantable cardiac monitoring devices. This review summarizes the current literature on AF burden and cardiovascular outcomes and outlines the effect and role of catheter ablation in ameliorating AF burden.

## 1. Introduction

Atrial fibrillation [AF] is a highly prevalent disease that represents a growing epidemiological problem. In 2010, the worldwide prevalence of AF was estimated to be around 33 million [1]. By 2019, the prevalence had almost doubled and was estimated to be around 60 million [2]. Of note, the age-related prevalence of AF appears to be stable in this study suggesting that the increased prevalence is attributable to longer life expectancy globally [2]. Contrasting data from the Framingham study showed an increase in age-related prevalence over the 50-year period studied [3]. Greater screening for AF and increased awareness have also contributed to the increase in prevalence [4].

AF has an important impact on health due to its effect on morbidity and mortality. AF increases the risk of stroke fivefold compared to the general population as shown in the landmark Framingham study [5]. AF is also a significant cause of cardiovascular mortality with an increasing trend. In the United States, the age-adjusted mortality rate increased from 18 per 100,000 in 2011 to 22 per 100,000 in 2018 [6]. AF also increases the risk of incident heart failure and complicates its management [7]. Even AF occurring in the post-operative setting is associated with significant consequences. This was shown in a meta-analysis of 28 studies that enrolled 2,612,816 patients, which demonstrated a threefold increase in stroke risk at one month and a fourfold increase in stroke risk at 12 months as well as a threefold increase in mortality risk [8]. On a similar note, AF detected during screening is also not benign. The community-based Prevention of Renal and Vascular End-stage Disease [PREVEND] study assessed 8265 participants without AF, with a mean follow-up of 10 years [9]. During this time, the incidence of AF was 3.2%, out of which 77% had developed clinical AF and 23% had AF detected during screening. AF detected though screening as well as clinical AF remained significantly associated with mortality and incident heart failure after multivariable adjustment [9].

AF burden is an interesting concept that has come to the fore in more recent times as an important modulator of AF outcomes [10]. Clinical assessment of the temporal burden of AF has been shown to be lacking accurate correlation to temporal persistence [11]. AF burden provides a quantitative measurement of AF and in its simplest terms is the amount of AF [10,12]. A consistent definition is needed for AF burden to allow direct comparisons of outcomes. AF burden can be measured in various ways such as the overall number of episodes or duration of the longest episode [13]. An important measure is the time spent in AF as a percentage of overall time monitored, but this is limited by the need for a long duration of monitoring such as that afforded by cardiac implantable electronic devices [14]. Device-detected AF has greatly contributed to our understanding of the relationship between subclinical AF and the risk of stroke [15]. The ideal monitoring duration has not yet been established. Shorter monitoring for 24–48 h using electrocardiogram monitors has been shown to be less sensitive for the detection of AF recurrence post-ablation and may overestimate AF burden in patients with AF recurrence [16]. In the same study, 28 days was found to be the optimal duration of monitoring needed to accurately establish AF burden. While implantable devices such as pacemakers and defibrillators are able to provide accurate measures of AF burden such as percentage of time in AF and the duration of each episode of AF, wearable devices are much more limited in their ability to accurately assess AF burden [10].

Catheter ablation is an important tool to address AF rhythm control, and due to recent evidence, there are expanding indications for this intervention. Compared to anti-arrhythmic drugs, ablation is more effective for maintenance of sinus rhythm [17]. This holds true when ablation is used as first-line therapy [18] and in complex patients such as those with heart failure [19]. Significant advances have been made in AF ablation with the introduction of new technology as well as expanding strategies beyond pulmonary vein isolation [20].

AF burden has significant consequences, and understanding the effect of AF ablation on cardiovascular outcomes and AF burden is necessary to comprehend its role in the management of AF. The impact of AF burden on stroke was recently reviewed [10] and is not the focus of this article. Here, we will review the current literature on AF burden and cardiovascular outcomes and summarize the effect and role of catheter ablation in ameliorating AF burden.

## 2. AF Burden and Cardiovascular Outcomes

AF burden has been associated with an increased risk of stroke. These findings were similar when examining the subclinical AF population, where greater burden in particular episodes greater than 24 h were associated with significant stroke [15], and clinical AF populations with more persistent forms of AF were associated with a greater stroke risk [10]. Beyond the risk of stroke, the impact of AF burden on cardiovascular outcomes was less clear.

An important association has been observed between AF burden and mortality and cardiovascular hospitalization. Chew and colleagues assessed data from over 39,000 patients who had paroxysmal AF and had received a new cardiac implantable electronic device to gauge the associations between AF burden as detected by cardiac implantable electronic devices and cardiovascular outcomes [21]. AF burden was defined as the daily percentage of time spent in AF ascertained at baseline over 30 days; another analysis based on the definition of maximal AF episode duration was also performed. Greater AF burden was associated with greater unadjusted cumulative incidence of mortality at 1 year: 8.5% in patients with an AF burden of 0%, 8.9% when daily AF burden was 0% to 5%, and 10.9% in patients with an AF burden between 5% and 98% (*p* < 0.001) [21]. AF burden was associated with an increased all-cause mortality risk at 1 year after adjusting for baseline characteristics; there was a 6% increase in mortality risk for every 10-percentage-point increase in daily AF burden [*p* < 0.001]. When the longest episode of AF exceeded ≥ 24 h, there was a 43% increase in the adjusted risk of all-cause mortality at 1 year [*p* < 0.001]. In summary, the authors found that increasing AF burden was independently associated with greater risks of all-cause mortality and cardiovascular hospitalization, at 1-year and 3-year follow-up with an exposure–response relationship. Interestingly, the association of AF burden and cardiovascular outcomes was similar when AF burden was measured either as the longest episode of AF or as the percentage of time in AF [21]. In a separate analysis, AF burden was significantly associated with all-cause mortality in both patients without underlying heart failure [5% increased risk per 10% AF burden] and with heart failure [6% increased risk per 10% AF burden] [22]. Therefore, it is now widely accepted based on multiple studies that a higher AF burden is an independent predictor of cardiovascular mortality [23].

Findings of an association between greater AF burden and increased mortality are also mirrored in secondary analyses of randomized controlled trials assessing the effectiveness of non-vitamin K oral anticoagulants. In the ROCKET-AF (Rivaroxaban Once Daily Oral Direct Factor Xa Inhibition Compared with Vitamin K Antagonism for Prevention of Stroke and Embolism Trial in Atrial Fibrillation) trial that compared rivaroxaban to vitamin K antagonists in patients with non-valvular AF, patients with paroxysmal AF had 21% lower all-cause mortality compared to patients with persistent AF [24]. The ARISTOTLE (Apixaban for Reduction in Stroke and Other Thrombo-embolic Events in Atrial Fibrillation) trial compared apixaban to warfarin in patients with non-valvular AF; patients with paroxysmal AF had 27% lower all-cause mortality than patients with persistent AF [25]. Analysis from the ENGAGE AF-TIMI 48 (Effective Anticoagulation With Factor Xa Next Generation in Atrial Fibrillation-Thrombolysis In Myocardial Infarction 48) demonstrated 27% and 22% lower all-cause mortality in patients with paroxysmal AF compared to patients with persistent AF and permanent AF, respectively [26]. The annualized all-cause mortality rate was higher in patients with persistent (4.4%/year; *p*-adj < 0.001) and permanent AF (4.4%/year; *p*-adj < 0.001) compared to patients with paroxysmal AF (3.0%/year) [26]. Finally, a meta-analysis of 12 studies which included around 100,000 patients and assessed all-cause mortality in patients with non-paroxysmal versus paroxysmal AF found that all-cause mortality was considerably higher with non-paroxysmal AF in both the unadjusted and adjusted analyses [27].

Other cardiovascular outcomes such as incident heart failure and heart failure hospitalization also follow a similar pattern. Analyses utilizing the aforementioned nationwide database with 39,710 patients and remote monitoring demonstrated that in patients without heart failure, a greater AF burden was associated with an increased risk of de novo heart failure [9% increased risk per 10% AF burden] [22]. In patients previously diagnosed with heart failure, there was an increased risk of heart failure hospitalization with greater AF burden [5% increased risk per 10% AF burden] [22]. Park and colleagues also investigated the association between AF burden and cardiovascular outcomes in patients with subclinical AF; the investigators quantified the burden of subclinical AF over a 6-month period using the total time spent in AF during every 6-month follow-up [28]. The median follow-up was 5.2 years, and 496 consecutive patients with pacemakers were classified into three groups based on AF burden: no AF burden if there were no AF episodes, low AF burden if the AF burden was less than 24 h, and high AF burden if the AF burden exceeded 24 h. The study showed a higher risk of myocardial infarction and heart failure hospitalization in the high-AF-burden group as well as a greater risk of cardiac death [28]. Higher AF burden is also associated with higher healthcare costs, largely driven by adverse cardiovascular outcomes requiring healthcare utilization [29].

**Figure 1:**
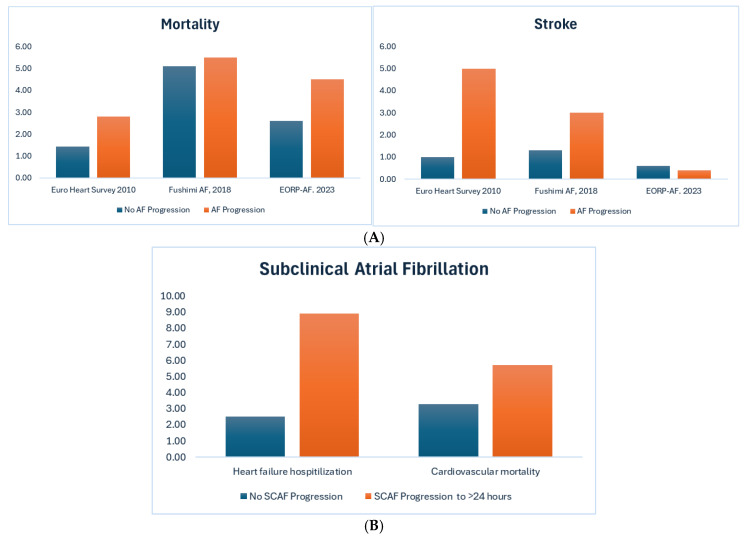
Atrial fibrillation progression and cardiovascular outcomes. Adapted from de Vos et al. 2010 [30], Ogawa et al. 2018 [31], Vitolo et al. 2023 [32], and Wong et al. 2018 [33]. AF = atrial fibrillation; SCAF = subclinical atrial fibrillation. (**A**) Progression versus no progression of paroxysmal AF; (**B**) progression of subclinical AF.

Progression to a higher AF burden has also been associated with adverse cardiovascular outcomes (Figure 1). In the EURO Heart survey in 2010, progression of AF from paroxysmal to persistent occurred in 15% of the 1219 patients included [30]. Predictors of progression included heart failure, age, previous transient ischemic attack or stroke, chronic obstructive pulmonary disease, and hypertension. Using these predictors, the HATCH score was conceived and validated to predict progression [30]. In this study, patients with AF progression had an increased risk of major adverse cardiovascular events and hospital admission [30]. The impact of progression to a higher burden of AF was also assessed using data from the Fushimi AF Registry, a community-based prospective survey of the patients with AF in Kyoto Japan that included 4045 patients. During the median follow-up period of 1105 days, progression of AF occurred in 252 [6.2%] patients with paroxysmal AF at a rate of 4.22 per 100 person-years [31]. AF progression was significantly associated with a higher risk of hospitalization for heart failure as well as an increased risk of ischemic stroke or systemic embolism and AF progression was a significant predictor for these events in multivariate regression analyses [31]. In another study, Vitolo and colleagues analyzed data from 2688 patients in the EURObservational Research Programme on Atrial Fibrillation [EORP-AF]. In the Long-Term General Registry, after a one-year follow-up period, 22.1% had progressed from paroxysmal to persistent AF [32]. At this point, patients were stratified into those who progressed and those who did not and followed-up for a mean 371 days; AF progression was independently associated with a higher risk for all-cause death compared to no-AF-progression patients [32].

Adverse events are not limited to progression of clinical forms of AF but extend to progression in subclinical AF. In an analysis of the ASSERT [Asymptomatic Atrial Fibrillation and Stroke Evaluation in Pacemaker Patients and the Atrial Fibrillation Reduction Atrial Pacing Trial] trial, the investigators identified patients who had subclinical AF between 6 min but less than 24 h and subsequently progressed to subclinical AF lasting longer than 24 h or clinical AF [33]. Over a 2-year follow-up period, 15% of patients progressed to episodes longer than 24 h or clinical AF at a rate of 8.8% per year, and these patients had an almost fourfold increase in the risk of heart failure hospitalizations compared to those who did not progress [8.9% vs. 2.5%] [33]. Multivariable analysis showed that progression of subclinical AF was independently associated with heart failure hospitalization [HR 4.58; 95% CI: 1.64 to 12.80; *p* = 0.004). Results were consistent when excluding patients with prior history of heart failure or limiting the definition of subclinical AF progression to only include those with episodes greater than 24 h and not those who progressed to clinical AF [33].

## 3. Ablation Effect on Burden and Progression

AF ablation has been demonstrated to be an effective therapeutic intervention for AF and is an important tool to reduce AF burden, reduce symptom burden, and enhance quality of life [34]. A recent analysis from the DECAAF II trial showed that ablation reduces AF burden, regardless of whether there is arrhythmia recurrence post-ablation. In this study, patients submitted daily single lead electrocardiogram strips, and AF burden was defined as the proportion of days in AF. The study also showed that left atrial fibrosis was an impediment to AF burden reduction post-ablation [35]. A strong correlation was also found between the observed reduction in AF burden and symptom improvement post-ablation [35]. Even patients who have been resistant to anti-arrhythmic drug therapy as well as electrical cardioversion can have a substantial reduction in AF burden after AF ablation. In one study assessing such patients who had their AF burden assessed by implantable monitors over a 12-month period post-ablation, 55% had no recurrence of AF and 45% had AF recurrence but with a 90% reduction in burden compared to pre-ablation [36]. AF burden was defined as the percentage of time in AF based on continuous monitoring by implantable monitors. The reduction in AF burden was also associated with quality-of-life improvement. These results support the use of AF ablation as a disease-modifying procedure that substantially decreases AF burden, despite the potential for recurrence of arrhythmia post-procedure. This may be explained by how the ablation procedure uses pulmonary vein isolation targets and eliminates AF triggers and modifies the arrhythmic substrate surrounding the pulmonary veins [37].

The effects of AF ablation on AF progression have also been assessed. In the ATTEST trial, which was a prematurely terminated randomized multicenter open-label trial, 255 patients with paroxysmal AF and a higher risk of progression of AF based on the HATCH score were randomized to either catheter ablation or anti-arrhythmic drug therapy and followed for three years [38]. The primary endpoint of persistent AF/atrial tachycardia at 3 years was significantly lower in those who underwent ablation (2.4% [95% CI: 0.6–9.4%]) versus those who received anti-arrhythmic drug therapy (17.5% [95% CI: 10.7–27.9%; *p* = 0.0009]) [38]. Proietti and colleagues analyzed the impact of AF ablation on progression of AF in a systematic review [39]. The percentage of AF progression at 1 year in the general AF population ranged from 10% to 20%. Progression in patients with heart failure was considerably higher (35% at 1 year). In the study with the longest follow-up period of 14 years, progression of AF occurred in 77%; progression of AF after catheter ablation was significantly lower [39]. Importantly, the effect of ablation is also observed in patients with recurrence after a previous ablation. In a randomized trial of 154 patients with paroxysmal AF who had recurrence of AF after a previous catheter ablation procedure with pulmonary vein isolation, they were randomized to either redo ablation or anti-arrhythmic drug therapy and were followed-up for 3 years by means of an implanted cardiac monitor. At the end of follow-up, 25% of patients who underwent redo ablation had progressed compared to 79% patients in the anti-arrhythmic drug group [*p* < 0.01] [40].

More recently, the impact of early AF ablation on progression has been assessed. The Early Aggressive Invasive Intervention for Atrial Fibrillation (EARLY-AF) trial, a randomized, multicenter open-label study with a blinded adjudicated endpoint, compared the use of catheter ablation with anti-arrhythmic drugs to prevent the recurrence of atrial tachyarrhythmia, which was assessed by an implantable rhythm monitor [18]. The trial randomized 303 patients with symptomatic paroxysmal AF to the treatment arms and followed them up for three years. During the three-year follow-up, 1.9% in the ablation group developed persistent AF compared to 7.4% in the anti-arrhythmic drug therapy group [hazard ratio 0.25; 95% CI, 0.09 to 0.70] [41]. Data from the CIRCA DOSE trial also showed that compared with that of the pre-ablation monitoring period, AF burden was reduced by a median 99% with both ablation strategies used with very few patients developing persistent AF at follow-up [42].

Benali and colleagues performed a meta-analysis of randomized controlled trials to assess the anti-progression effect of catheter ablation on paroxysmal AF as well as the effect of intervention timing [43]. The authors included trials that compared AF catheter ablation to anti-arrhythmic drug therapy in patients with paroxysmal AF and assessed the rate of progression to persistent AF at 3 years of follow-up; five randomized trials met these inclusion criteria. The rate of progression to persistent AF at three years was 8.3% [95% CI 5.4–11.2]. Progression is affected by age and comorbidities such as those identified by the HATCH score. In the EARLY-AF trial, the observed rate of progression was 1.9% after cryoballoon ablation which reflects the lower risk in the included population [41]. In the ATTEST trial which used radiofrequency, the risk of progression was slightly higher at 2.4% [38]. In the CIRCA-DOSE trial which included both modalities, the risk of progression with cryo-ablation was higher than that observed in EARLY-AF at 5.6% but the progression after radiofrequency ablation was lower than that observed in ATTEST at less than 1% progression [42]. In the meta-analysis by Benali and colleagues, the rate of progression to persistent AF at three-year follow-up was significantly lower in patients randomized to AF ablation [1.8%; 95% CI 0.3–3.3] than those who received anti-arrhythmic drugs [14.9%; 95% CI 9.3–20.5] with an 85% lower relative risk [0.15; 95% CI 0.08–0.28; *p* < 0.001] for AF ablation. The relative risk reduction in progression of AF was similar whether ablation was the first line (81%) or not the first line (87%) [43]. In summary, the risk of progression from paroxysmal to persistent AF was reduced by sevenfold at three years with catheter ablation compared to anti-arrhythmic drug therapy, regardless of the timing of ablation, with a number needed to treat of eight to prevent one progression [43].

## 4. Ablation to Reduce Cardiovascular Outcomes

**Figure 2:**
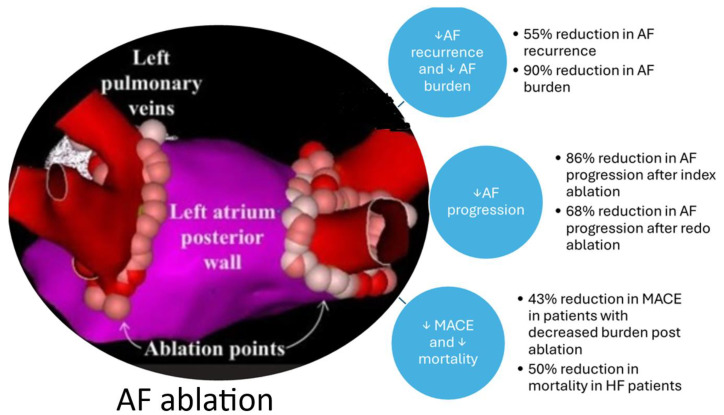
Effect of ablation on atrial fibrillation burden and cardiovascular outcomes. Adapted from AlTurki et al. [19], O’Neil et al. [36], Kuck et al. [38], Pokushalov et al. [40] and Bunch et al. [46]. AF = atrial fibrillation; HF = heart failure; MACE = major adverse cardiovascular events.

Considering the significant effect of AF burden on cardiovascular outcomes, patients may benefit from attempts to lower AF burden through AF ablation (Figure 2). Several studies have shown a reduction in adverse cardiovascular outcomes with AF ablation, which is likely mediated by lower AF burden post-ablation. While the landmark AFFIRM trial showed no difference between rhythm control and rate control medication with regard to cardiovascular outcomes, further analyses demonstrated that patients who remain in sinus rhythm had lower overall mortality [44]. The Catheter Ablation vs. Anti-arrhythmic Drug Therapy for Atrial Fibrillation trial [CABANA] trial was a large, investigator-initiated, multicenter, open-label randomized trial that compared AF ablation to medical therapy in 2204 symptomatic patients with AF aged 65 years and older or younger than 65 years with one or more risk factors for stroke [45]. The intention to treat analysis showed no difference in cardiovascular outcomes between catheter ablation and medical management but the trial had significant challenges including a high crossover rate from medical therapy to catheter ablation and a low overall event rate for cardiovascular outcomes. In a time-dependent analysis of the CABANA trial, low AF burden as evidenced by the presence of sinus rhythm was associated with a significantly reduced risk of the primary endpoint of death, disabling stroke, serious bleeding, or cardiac arrest [adjusted HR, 0.57; 95% CI, 0.38–0.85; *p* = 0.006] [46]. The reduction in adverse cardiovascular outcomes due to AF ablation is also observed in “real-world” data. In a study of 3084 patients who underwent AF ablation using data from a large administrative database and using propensity-matching, AF ablation was associated with a reduction in overall mortality and cardiovascular hospitalizations [47].

Heart failure is an important contributor to adverse cardiovascular outcomes in AF patients and vice versa; adverse cardiovascular outcomes occur at a significantly higher rate in AF patients with heart failure than those without. Heart failure hospitalizations are serious cardiovascular events that signal an impending risk of cardiovascular mortality [48]. AF ablation has been shown to reduce cardiovascular outcomes in patients with AF and heart failure including mortality and heart failure hospitalizations. In a meta-analysis of seven randomized controlled trials, enrolling 856 patients, AF ablation as compared with medical therapy was associated with a significant reduction in mortality [risk ratio 0.50; 95%CI: 0.34 to 0.74; *p* = 0.0005] and heart failure-related hospitalizations (risk ratio 0.56; 95% CI: 0.44 to 0.71; *p* < 0.0001) [19]. The Catheter Ablation versus Standard Conventional Therapy in Patients with Left Ventricular Dysfunction and Atrial Fibrillation (CASTLE-AF) trial was the largest trial in this population. CASTLE AF randomized 363 patients with symptomatic paroxysmal or persistent AF who had failed medical therapy to undergo either AF ablation or medical therapy for atrial fibrillation while continuing heart failure treatment [49]. The primary endpoint, which was a composite of death from any cause or hospitalization for worsening heart failure, was significantly lower after catheter ablation [28.5%] than medical therapy [44.6%] after a median follow-up of 37.8 months [HR 0.62; 95% CI, 0.43 to 0.87; *p* = 0.007]. When looking at individual outcomes, all-cause mortality [HR 0.53; 95% CI, 0.32 to 0.86; *p* = 0.01], hospitalizations for worsening heart failure [HR 0.56; 95% CI, 0.37 to 0.83; *p* = 0.004], and cardiovascular mortality [HR 0.49; 95% CI, 0.29 to 0.84; *p* = 0.009] were all lower in the ablation group compared to the medical therapy group [49]. In a post hoc analysis of the CASTLE AF that assessed the effect of AF burden defined as the percentage of time in AF based on implantable devices, an AF burden that was less than 50% six months after catheter ablation, was associated with a significant decrease in primary composite outcome [HR 0.33; 95% CI: 0.15 to 0.71; *p* = 0.014) and all-cause mortality (HR: 0.23; 95% CI: 0.07 to 0.71; *p* = 0.031). Importantly, the risk of mortality was directly related to a low (<50%) or high (≥50%) AF burden at 6 months post-ablation [50].

Early ablation also appears to be beneficial in heart failure and reduces AF burden [51]. Sakamoto and colleagues also assessed patients who were hospitalized for acute decompensation for heart failure and had AF. Early AF ablation within 90 days of admission was associated with lower all-cause mortality (HR: 0.38, 95% CI: 0.24–0.60; *p* < 0.001) as well as lower cardiovascular death and heart failure-related death with similar results in matched analyses [52]. In a meta-analysis of twenty-two randomized trials, there was a reduction in adverse cardiovascular outcomes such as cardiovascular mortality [53]. Sensitivity analyses showed that these benefits were driven by trials in heart failure patients. However, the analyses also showed that there was potentially more benefit in patients with persistent AF or more pronounced AF burden [53].

Importantly, most of the studies that demonstrated a benefit for AF ablation were in patients with symptomatic AF. The evidence for patients with truly asymptomatic AF is insufficient to recommend ablation. This is in keeping with current guidelines which state that only highly selected asymptomatic patients could be candidates for AF ablation given the associated risks as well as the potential benefit that may be derived from delaying AF progression [54].

## 5. Future Directions

There remain several unanswered questions in the connection between the burden of AF and cardiovascular outcomes as well as the role of catheter ablation. These include a threshold for AF burden for interventions such as anticoagulation and catheter ablation. The latter is particularly important given the benefits mentioned of early rhythm control. Implantable and wearable monitoring devices will increase detection, and more accurate determination of AF burden and studies will be needed to address the implications of treatment. Furthermore, imaging parameters such as strain imaging and atrial fibrosis may be able to predict patients at risk of a higher burden of AF. A consensus definition is also needed for AF burden to facilitate further research and incorporation into clinical practice guidelines. Further studies are also needed to assess the effects of preventing or slowing the progression of AF especially through use of catheter ablation to reduce AF burden. More studies are also needed in groups that have been poorly represented in studies such as females and non-White ethnicities. Studies on catheter ablation of AF should also use AF burden when measuring recurrence given the correlation with adverse cardiovascular outcomes compared to the arbitrary cut-off of 30 s which does not correlate with outcomes. Studies are needed to determine an AF burden cut-off that correlates with lower quality of life and adverse cardiovascular outcomes. There are two ongoing trials that will assess the role of AF ablation in outcomes in patients with AF and HFpEF (CABA-HFPEF, NCT05508256) [55] and in patients with AF and multiple comorbidities (EASThigh-AFNET 11, NCT06324188) by reducing AF burden. These trials will further delineate the impact of reducing AF burden through AF ablation on cardiovascular outcomes. Asymptomatic AF is an area that requires further research as most studies that assessed cardiovascular outcomes included patients with symptomatic AF. In addition, asymptomatic AF was found to account for 27% of the AF population [56]. In a recent large meta-analysis, the risk of cardiovascular death and stroke was found to be independent of the presence of AF symptoms [57]. The study also showed that patients with asymptomatic AF were less likely to receive interventions for rhythm control and more likely to progress to a higher AF burden [57]. Therefore, studies are needed to show the effect of early intervention in these patients, particularly those with a higher AF burden.

## 6. Conclusions

There is now clear evidence demonstrating the association between greater AF burden and adverse cardiovascular outcomes such as mortality, incident heart failure, and heart failure hospitalizations. In addition, progression of AF burden is also associated with adverse cardiovascular outcomes. Catheter ablation considerably reduces the burden of AF and attenuates the progression of AF. Furthermore, AF ablation has also been shown to reduce AF burden and reduce adverse cardiovascular outcomes including mortality, especially in patients with heart failure.
